# How Do Green Innovation Strategies Contribute to Firm Performance Under Supply Chain Risk? Evidence From China’s Manufacturing Sector

**DOI:** 10.3389/fpsyg.2022.894766

**Published:** 2022-04-28

**Authors:** Mengmeng Wang, Zhaoqian Liu

**Affiliations:** College of Business, Gachon University, Seongnam-si, South Korea

**Keywords:** sustainability, green innovation, green product innovation, green process innovation, green service innovation, firm performance, supply chain risk

## Abstract

With environmental issues increasingly becoming prominent in today’s business world, firms may need to pay extra attention to developing their environmental strategies and capabilities in response to environmental concerns and achieving sustainable growth. While a broad consensus exists on the value of green innovation, current empirical research on how different types of green innovation strategies may account for the international performance of a firm remains scant. Addressing this gap is important because determining how to better manage a firm’s green innovation strategies nowadays has become increasingly important for firms hoping to achieve and maintain their sustainable performance advantages. This study aims to bridge this gap by systematically examining how various types of green innovation strategies (i.e., green product, green process, and green service innovations) can be beneficial to firms in an emerging market economy. This study also examined the important role that potential risks of supply chain play in shaping the relationships between various types of green innovation strategies and firm performance. This study proposes that the effective management of supply chain risks may be important to the successful implementation of green innovation strategies because green innovation has increasingly become a collaborative effort. This study empirically tested the hypotheses by gathering survey data from a sample of 337 firms in China’s manufacturing industries. Results demonstrate that the green innovation strategies of firms are positively related to their firm performance. Additionally, the potential risks faced by the firms in efficiently and effectively managing their supply chain significantly moderate the impact of green product innovation and green process innovation strategies on their firm performance. This study not only offers useful theoretical implications for the green innovation strategy research and for better and effective supply chain risk management. It also provides important practical guidelines and managerial actions that practicing managers can implement to accelerate their green innovation strategy, assess the effect of supply chain risks, and thus improve firm performance in the post-pandemic era.

## Introduction

Sustainable development is an important topic in the twenty-first century, and green innovation is an important driving factor to achieve sustainable development. With sustainable development and continuous popularization of the concept of green environmental protection, green innovation has become an important direction of firm development. Faced with growing environmental problems, many countries have established environmental regulations to regulate and reduce pollution and damage to the environment in production and service processes. With the continuous growth of their environmental awareness, consumers are placing increasing value on the green attributes of a product (Mu et al., 2021). Green innovation is essential for the sustainable development of a firm and the satisfaction of consumers. Green innovation includes hardware and software innovation. It is achieved by adopting eco-friendly materials, developing energy-saving technologies, and reducing processes (Schiederig et al., 2012). Specifically, green innovation can be divided into three parts: green product innovation, green process innovation, and green service innovation.

Green product innovation includes innovations in product design to reduce environmental impacts during production, use, and disposal at the end of the product’s life (Arfi et al., 2018). It focuses on the use and recycling of eco-friendly materials to reduce material waste and energy in the production process (Khan et al., 2021). Compared with traditional product innovation, green product innovation is an innovation undertaken by companies to meet environmental changes and customer expectations by reducing excessive consumption of raw materials and energy to avoid risks to consumer health and safety (Chen et al., 2017). This innovation focuses on environmental issues, emphasizes corporate environmental responsibility, and places importance on the use and disposal of products, including energy conservation, pollution prevention, waste recovery, toxicity reduction, and environmental design (Chen et al., 2006; Tariq et al., 2017). Green product innovation meets the needs of consumers for environmental protection, helps firms develop new markets, makes copying of products difficult for other firms, and maintains product competitiveness. Successful green product innovation not only can improve resource utilization efficiency but also enable firms to obtain a competitive advantage (Dangelico and Pujari, 2010; Chang, 2016; Andersén, 2021).

Green process innovation has been widely recognized by governments, scientific research organizations, and social groups (Dugoua and Dumas, 2021). As one of the most basic elements of green innovation and a necessary explicit requirement for implementing green product innovation, green process innovation emphasizes the innovation of production process by using the approaches such as the introduction of advanced green process, green production equipment, and green recycling methods to minimize environmental load (Ma et al., 2017). Compared with traditional innovations, green process innovations play an irreplaceable role in improving environmental quality to reduce environmental pollution and energy and raw material consumption (Guo et al., 2020). This innovation incorporates the environmental needs of stakeholders into production design by reducing the cost of producing goods and aligning products with environmental regulations (Hart and Dowell, 2011). It applies the concept of green to the entire process of product innovation by increasing resource utilization, efficiently promoting green production design, and positively promoting corporate financial performance (Xie et al., 2019). Accordingly, businesses can reduce their environmental and operating costs while indirectly improving their economic performance (Ma et al., 2021). Green process innovation has a positive effect on the reputation, image, and economic performance of a firm (Lee and Min, 2015). It can help develop green products, enlarge product size, improve product quality, raise the reputation of the firm, increase its market share, and realize sustainable development (Xie et al., 2019).

Green service innovation includes elements such as green invention, environmental service portfolio, environmental service delivery, and environmental service design (Chen et al., 2015). Distinct from other service innovations, green service innovation focuses on environmental social responsibility and customer experience (Vakulenko et al., 2019). It is a unique service that rivals will not easily replicate by primarily considering the environmental impact of services provided by companies (Lin and Chen, 2017). In the course of green service innovation, the company repackages new products and services according to environmental concerns, promises environmentally friendly sales practices and after-sales services, and actively helps companies achieve their sustainable development goals (Chen et al., 2015). Companies gain the upper hand by promoting green service innovation activities, such as green services, green design, and clean production (Chuang and Huang, 2015). They can also increase entry barriers to rivals through green service innovation (Chang, 2018).

Based on the above analysis, green product innovation, green process innovation, and green service innovation are the inevitable trends of future development. They can bring many benefits to firms, but challenges and opportunities coexist. Some scholars believe that firms need to invest considerable resources and equipment and a certain amount of funds for waste treatment to implement green innovation. Energy conservation and emission reduction through a green treatment process will increase production cost. The cost of producing green products is significantly higher than that of producing similar non-green products, the profit space is largely reduced, and the competitiveness of product price is not high, all of which will have a negative impact on and cause difficulty in improving the firm’s market performance (Palmer et al., 1995; Ambec and Lanoie, 2008; Bray et al., 2011; Wong et al., 2012; Olson, 2013). A look at previous research on the impact of green innovation on firm performance reveals that academia has different views on whether green innovation strategy can have a positive impact on firm performance. Apart from using different empirical settings and research samples, another important plausible explanation for the conflicting findings is that these prior studies tend to view green innovation as a whole and thus do not demarcate specific types of green innovation (e.g., green product innovation, green process innovation, and green service innovation). With the increasingly prominent contradiction between environmental protection and economic development, more and more firms hope to break through the bottleneck and improve their performance through green innovation. In this regard, we recognize the urgency of adding to our understanding of important green innovation issues by demarcating specific types of green innovation, and more importantly, by identifying and exploring how specific types of green innovation contribute to firm performance. At this time, research on which types of green innovation firms improve their performance is extremely important. To fill the gap in previous research, this study takes the exploration of and analysis on the impact of green innovation on firm performance as the main research themes. First, green innovation is divided into three parts, namely, green product innovation, green process innovation, and green service innovation. Additionally, empirical analysis is used to verify green product innovation. By investigating the different effects of green process innovation and green service innovation on firm performance, we hope to lay the foundation for follow-up green innovation research, deepen and expand the research scope of green innovation and firm performance, help firms deepen their understanding of green innovation strategy, and provide another theoretical basis for firms to implement green innovation strategies.

Under the current background of global economic integration and globalization of environmental issues, a continuing effort has been made by firms to improve their performance by implementing green innovation. In this situation, considerable attention has been increasingly given to the issue of supply chain risks. For example, the question of whether upstream firms can guarantee the adequate supply of important equipment needed to treat wastewater, gaseous wastes, and other types of wastes becomes a genuinely big issue. Therefore, this paper presents a resolution to the debate, by investigating whether and how supply chain environmental conditions such as risks moderate the specific type of green innovation and firm performance. With deepening globalization, future competition will no longer be between firms but between supply chains instead. In the process of global firms adapting to environmental changes and adopting green innovation strategies, the upstream and downstream firms in the supply chain are closely related. Mistakes in any part of the chain may cause considerable losses to the whole supply chain. If the upstream side cannot supply in time, it will lead to the interruption of downstream transportation and in turn, the whole supply chain. Hence, supply chain risk cannot be ignored (Cao and Zhang, 2011). Supply chain risk is the risk that some uncertain factors or emergencies have a negative impact on one or more supply chain links, resulting in the reduction of supply chain efficiency and even the interruption of the supply chain. Its uncertainty is reflected in the inability of firms to accurately judge when, where, in what form, and to what extent the risk will cause losses to upstream and downstream firms (Heckmann et al., 2015). Any risk factor affecting the delivery of products from suppliers to end users can be defined as supply chain risk (Peck, 2006). Supply chain risk generally includes demand interruption and supply interruption. Demand interruption refers to the reduction of market demand because of emergencies. Supply interruption refers to the supplier’s failure to achieve supply within the commitment period (Shen and Li, 2017). The sudden pandemic has slowly become long term, and mankind has paid a huge economic and social price. Supply chain risks such as border blockade, sea and air transportation interruption, and logistics obstruction emerged one after another, and firms are forced to deal with them. An increasing number of suppliers are unable to supply goods according to their contract, and the uncertainty of supply and demand has increased. During the pandemic, global trade protectionism increased, some countries strengthened the protection of their firms and markets, the global internationalization process slowed down, market uncertainty and complexity increased, and more and more risk factors appeared in the supply chain. Owing to the close relationship between upstream and downstream firms in the supply chain, if the upstream cannot supply in time, it will lead to the interruption of downstream transportation. Errors in any link of the supply chain will cause considerable losses to each firm, and each firm in the supply chain is not alone (Cao and Zhang, 2011). In view of the fact that firms need to fully control supply chain risks in the process of green innovation practice, the present study takes understanding and grasping the moderating role of supply chain risks as the second research purpose. From the perspective of supply chain risks, this study explores and analyzes the moderating role of supply chain risks between green innovation and firm performance to help firms avoid supply chain risks in the process of green innovation while providing effective solutions for firms to maximize their performance.

## Theoretical Background and Hypothesis Development

The theory of circular economy was first mentioned by British environmental economists David Pearce and Kerry Turner in the book, “Natural Resources and Environmental Economics” (Pearce and Turner, 1990). In the face of existing environmental problems and resource shortage, this theory states that the relationship between economy and the environment is circular (Li and Liu, 2010). The most important principles of a circular economy are reduction, reuse, and recycle. Reduction means minimizing the input of primary energy and raw materials by improving production efficiency. Reuse means recommending the use of by-products and waste of one company as resources for other companies or industries to maximize product utilization. Recycling encourages the processing of recyclable materials into new products to reduce the consumption of raw materials. Given the different levels, the three principles have different functions and degrees of importance in the economy. Among them, reducing resources is the leading principle in the circular economy system (Su et al., 2013).

**FIGURE 1:**
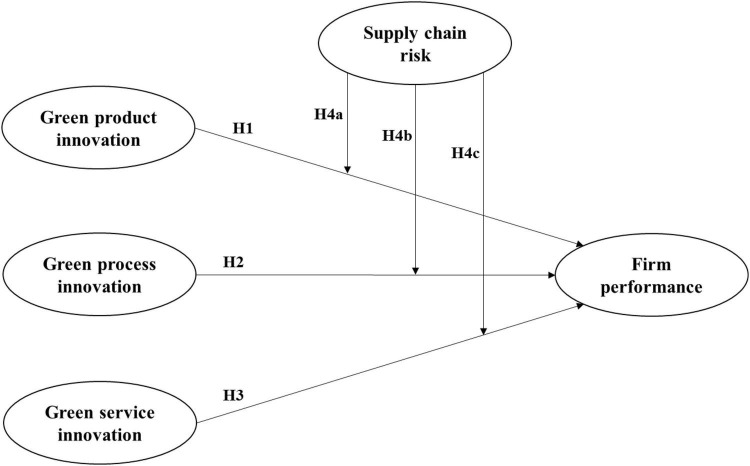
Conceptual model.

Figure 1 illustrates the research model inspired from the theory of circular economy and proposed in this study. As shown in Figure 1, this study divides green innovation into green product innovation, green process innovation, and green service innovation. Hypotheses 1–3 propose that green product innovation, green process innovation, and green service innovation, respectively, have different positive promoting effects on firm performance. At the same time, in view of the changes in the international situation and based on the original circular economy theory, taking the global supply chain risk as a moderating variable, Hypotheses 4a–4c assume that supply chain risk negatively moderates the contribution of green product innovation, green process innovation, and green service innovation to firm performance, respectively.

### Green Product Innovation and Firm Performance

According to previous literature research, green product innovation reduces the negative impact of firms on the environment and improves the profitability of the firms by reducing waste and costs (Singh et al., 2020). Green product innovation also plays a positive role in promoting the establishment of corporate image (Weng et al., 2015). With the continuous improvement of consumers’ awareness and demand for environmental protection, firms are providing more environmentally friendly products than their competitors, which help the former develop new markets and gain competitive advantages (Chen et al., 2006). The implementation of green product innovation by firms enhances the possibility of implementing differentiation strategies and helps improve consumers’ perceived value of products (Porter, 1991). In view of the benefits of green product innovation, its implementation is anticipated to have a positive impact on firm performance in the market competition. On this basis, this study proposes the following relationship:

*Hypothesis 1*: A firm’s level of green product innovation is positively associated with the firm’s performance.

### Green Process Innovation and Firm Performance

From the perspective of innovation economics, green process innovation can improve the economic performance of firms by optimizing the factor allocation efficiency of firms, including reducing production and operation costs, expanding production, increasing market share, obtaining green technology patent license, and other benefits (Wang et al., 2021). Green process innovation plays a positive role in improving resource utilization and reducing input and waste treatment costs (Wei and Sun, 2021). Given the increasingly serious problem of global environmental degradation, governments of various countries have formulated and issued a series of environmental protection policies to restrict the production of pollutant discharge firms. The pressure on a firm to practice green process innovation is increasing. As an important part of green innovation, green process innovation can significantly reduce pollution in the manufacturing process and meet the green needs of the government and consumers (Dai and Zhang, 2017). With the continuous enhancement of consumers’ awareness of environmental protection, they not only like to buy energy-saving and environmental protection products but also want to know whether a green process is used in the production process of the products. Environmental protection products through green process innovation can be highly praised by consumers (Liu et al., 2012). To sum up, firms may improve their performance if they implement green process innovation in market competition. On this basis, this study proposes the following relationship:

*Hypothesis 2*: A firm’s level of green process innovation is positively associated with the firm’s performance.

### Green Service Innovation and Firm Performance

In green firms, customer demand is the key to whether firms can improve performance, and it can be fully met by providing green service innovation (Yu et al., 2017). Green service innovation focuses on environmental issues and can create unique services that cannot be easily copied by competitors (Chen et al., 2016). Such innovation provided by firms not only reduces the negative impact on the environment but also helps firms integrate with the international market and better meet the environmental requirements of the international community (Lin and Chen, 2018). Green service innovation is a process to attract customers by improving the environmental protection of products and services, thus providing customers with green experience and helping firms improve their market share (Yu et al., 2017). It can add value to products and services as well as improve the competitiveness and creativity of firms to help them gain competitive advantage (Lin and Chen, 2017). Overall, providing green service innovation may contribute to firm performance. On this basis, this study proposes the following relationship:

*Hypothesis 3*: A firm’s level of green service innovation is positively associated with the firm’s performance.

### Moderating Effect of Supply Chain Risk

Generally, inaccuracy in predicting market demand will lead to excess inventory and increase in storage costs. Drastic changes in the international environment will lead to fluctuations in raw material prices and increase in procurement costs. Natural and man-made disasters will affect production and interrupt the supply chain. Firms are said to be deep in the supply chain, and any uncertain factors and negative events may affect their performance (Kuo and Lin, 2018). The firm supply chain itself is fragile. Affected by factors such as firm outsourcing, the emergence of the global market, increasing dependence on suppliers and customers, and the rapid development of information technology, supply chain risks are increasing in the process of implementing an innovation strategy (Kuo and Lin, 2018). Once supply chain risks reach the degree of interruption, the downstream supply will be unstable and unable to respond to the needs of customers immediately, and the firm performance will suffer losses (Sheffi, 2001; Hendayani et al., 2021). As the biggest uncertainty in the sustainable development of firms, environmental problems have a huge impact on firm performance. If firms cannot control the supply chain risks well, these risks may have a negative impact on firm performance (Singh, 2020). Firms have promoted green product innovation, green process innovation, and green service innovation to stabilize firm performance and thus prevent the uncertainty of environmental problems and reduce the supply chain risks caused by environmental problems. To reduce the negative impact on the environment, green product innovation uses decomposable materials for product packaging, designs environmentally friendly packaging for products, establishes the environmental image of the firm, and reduces the supply chain risk caused by the uncertainty of environmental problems (Chen et al., 2006). Green process innovation means that firms improve the product process environment; reduce pollutants or harmful substances in the production process; prevent firms from polluting the soil, water quality, noise, and air in the production process; and recycle waste, wastewater, and resources (Chen et al., 2006; Xie et al., 2019). Green service innovation means that firms provide green services for customers on the basis of environmental concerns and social responsibility, make environmental protection commitments for environmental problems, provide environmental protection sales methods and after-sales services, and actively strive to practice environmental protection practices (Chen et al., 2015). Green innovation strategy applies the green concept to the whole process of product innovation, which can improve resource utilization efficiency, effectively promote the production and design of green products, improve product quality and firm popularity, increase market share, achieve sustainable development, and positively promote firm performance (Lee and Min, 2015; Xie et al., 2019). When firms use the green innovation strategy to expand overseas, opportunities and challenges coexist; moreover, they may encounter political, social, environmental, supplier, and customer supply chain risks (Brusset and Teller, 2017). Owing to different environmental regulation standards in various countries, governments, suppliers, and customers have different views on environmental issues (d’Astous and Legendre, 2009; Wu and Ma, 2016). The degree of supply chain risk encountered by each firm is also different. Thus, the following are expected: the greater the supply chain risk, the more obstacles for firms to practice green innovation; and the lower the supply chain risk, the smoother the path of firm green innovation and the higher the firm performance. Taken together, this line of reasoning suggests that, in most circumstances, supply chain risks weaken the contribution of green innovation to firm performance. Thus, any given firm experiencing greater supply chain risks is more likely to achieve worse performance than the firm experiencing less supply chain risks by implementing green product innovation, green process innovation, and green service innovation on firm performance. On this basis, we propose the following relationships:

*Hypothesis 4a*: The level of supply chain risk negatively moderates the relationship between green product innovation and the firm’s performance, such that the higher the degree of supply chain risk, the lower the contribution of green production innovation to firm performance.

*Hypothesis 4b*: The level of supply chain risk negatively moderates the relationship between green process innovation and the firm’s performance, such that the higher the degree of supply chain risk, the lower the contribution of green process innovation to firm performance.

*Hypothesis 4c*: The level of supply chain risk negatively moderates the relationship between green service innovation and the firm’s performance, such that the higher the degree of supply chain risk, the lower the contribution of green service innovation to firm performance.

## Methodology

### Sampling and Data Collection

We test our hypotheses using survey data collected from a sample of firms in China’s manufacturing sector. China provides an appropriate research setting to empirically explore how the type of green innovation may determine firm performance and how supply chain risk may moderate the contribution of the type of green innovation to firm performance. With the quick innovation-driven transformation, China has been making active efforts to boost innovation in manufacturing by speeding up and upgrading the green technological innovation, particularly the greening of its manufacturing system to enhance global competitiveness and achieve a more sustainable high-quality development in the long run. Accordingly, many Chinese firms are actively seeking innovation in products, services, business models, and core technologies by further ramping up their efforts in core technologies to foster green and sustainable development. According to the 2021 China manufacturing innovation survey report on large- and medium-sized manufacturing firms released by Deloitte Consulting, more than 30% of the surveyed Chinese manufacturing firms have been engaged in various innovation activities such as product, service, or technological innovation; meanwhile, 9% of the firms have been seeking more green-oriented innovation (Deloitte Consulting, 2021). The report also indicated that Chinese firms generally experience many persisting significant challenges in boosting green and sustainable innovation. Furthermore, as China has announced that its carbon emissions will be expected to peak its carbon emission before 2030 and achieve carbon neutrality before 2060, the Chinese government has been introducing a series of supporting policies and incentives to encourage and facilitate Chinese firms to deploy innovative technologies that will speed up their green transformation.

We followed a careful process to develop the questionnaire for the study. We first developed an English version of the questionnaire and then translated it into Chinese by two independent bilingual translators. To ensure conceptual equivalence and accuracy, the Chinese version of the questionnaire was back-translated into English by two additional independent bilingual professional translators. Prior research has pointed for the difficulties of collecting sufficient primary data from Chinese firms and emphasized the particular importance of developing a good relationship and trust with the sampling firms to increase high-quality responses (Hoskisson et al., 2000). Therefore, we conducted the survey procedures with the help of a renowned research company in the Chinese local market. We received a total of 353 questionnaires. After excluding 16 incomplete responses, we received a total of 337 completed and usable questionnaires that are utilized for the final data analysis. Responding firms operating primarily in industrial markets accounted for 62%. The participating firms with annual sales less than 5 million RMB accounted for 31.5 and 20.5% of the participating firms ranged in size from 5 million RMB to 10 million RMB in annual sales. Regarding ownership structure, nearly 63% of the responding firms were privately owned enterprises.

To check for the presence of non-response bias in the survey data that may influence our statistical results, we compared the differences between the responding firms and non-responding firms as well as the early- and late-responding firms, and the results of such comparison demonstrated that these groups did not differ statistically in terms of key firm characteristics (e.g., firm size). We also checked for the presence of potential common method variance (CMV) in our data. Following Podsakoff et al. (2003), we assessed the potential CMV concern in our data by performing Harman’s one-factor analysis. Accordingly, we performed exploratory factor analysis by running non-rotated factor analysis with all multiple-item variables entered. The results of the one-factor analysis indicate that no general factor is apparent in the unrotated factor structure and accounts for a majority of the variance, thereby suggesting that CMV is less likely to be a significant concern in our data.

### Variables and Measurement

Unless noted otherwise, we measured all the dependent, independent, and moderating variables in the study using multiple-item, seven-point Likert scales ranging from “strongly disagree” (1) to “strongly agree” (7).

The dependent variable, firm performance, represents the degree of self-reported performance. Following prior research (e.g., Katsikeas et al., 2006; Schilke, 2014; Park and Xiao, 2020), we measured firm performance by asking the firms to assess their profitability, net profit margin, profitability growth, sales performance, and overall firm performance compared with those of their industry rivals. To measure a firm’s green product innovation, we used four items derived from prior related research (e.g., Chen et al., 2006; Lin et al., 2013; Xie et al., 2019). On the basis of the work of Xie et al. (2019) and Wang et al. (2021), we measured green process innovation using eight items. To measure green service innovation, we used five items derived from prior literature (e.g., Chen and Tsou, 2006; Chen et al., 2015). Following prior research (e.g., Brusset and Teller, 2017), we measured the degree of supply chain risks using six items.

To rule out alternative explanations for our results, we also incorporated several control variables into the analysis: firm size, industry type, and ownership structure. We included firm size measured as a firm’s annual sales (Qian et al., 2010; Zhao and Murrell, 2016). We controlled for the industry effect using a dummy variable, which was equal to 1 if the firm’s product domain was industrial (Takata, 2016). To control for the effect of ownership structure, we developed a dummy variable which was equal to 1 if the firm is privately owned (Houston et al., 2011; He et al., 2013).

## Analyses and Results

### Measure Reliability and Validity Assessment

Before empirically testing the hypotheses, we first assessed the reliability and validity of the constructs. Table 1 presents the results of the reliability and validity assessment, which summarizes the construct reliabilities, factor loadings, and the average variances extracted (AVEs). As we used the established scales to measure the variables in this study, all measures exhibit strong reliability and validity. As shown in Table 1, all the Cronbach’s alpha values, ranging from 0.913 to 0.957, are greater than 0.90, exceeding the 0.70 benchmark. Therefore, our constructs exhibit strong internal reliability (Nunnally, 1978). In addition, we assessed the construct validity using confirmatory factor analysis (CFA). The fit indexes of the CFA analysis show that the overall model offers satisfactory fit to the data [χ^2^/df = 2.09, *p* < 0.001; comparative fit index (CFI) = 0.959; Tucker Lewis Index (TLI) = 0.954; incremental fit index (IFI) = 0.959; root mean square error of approximation (RMSEA) = 0.057]. The factor loadings of all constructs are highly significant with values greater than 0.70. The composite reliability of all constructs, ranging from 0.917 to 0.957, exceeds the 0.70 benchmark and all AVE values, ranging from 0.651 to 0.789, are greater than 0.50. These results provide adequate reliability and convergent validity (Fornell and Larcker, 1981). Following Fornell and Larcker (1981), we assessed discriminant validity of the measures by comparing the square root of AVE of each construct and correlation between the construct and all possible pairs of constructs in the model. As shown in Table 2, the results confirmed that the square root of AVE of each construct is much higher than its correlation with the other constructs, providing an adequate discriminant validity of the measures. Overall, the constructs and their respective indicators exhibit strong reliability and validity in the context of this study.

**TABLE 1:** Results of reliability and validity assessments of the constructs.

Construct and indicators	FL
***Green product innovation* (Cronbach’s alpha = 0.913, *CR* = 0.917, AVE = 0.736)**	
Modifications of product design not to use toxic compounds within the production process.	0.861
Product design reformations aimed to improve energy efficiency during usage.	0.887
Product packaging with decomposable materials for lower disposal environmental impact.	0.889
Improving and designing environmentally friendly packaging for existing and new products.	0.790
***Green process innovation* (Cronbach’s alpha = 0.937, *CR* = 0.937, AVE = 0.651)**	
The environmental improvement of products reduces pollutants or hazardous materials within the production process.	0.755
The environmental improvement of the product has reduced soil, water quality, noise, and air pollution within the production process.	0.743
The environmental enhancement of the product leads to the recycling of waste, water, and materials within the production process.	0.801
The environmental enhancement of the product leads to a reduction in energy use within the production process.	0.828
The environmental contribution of the product leads to reduced soil, water quality, noise, and air pollution within the production process.	0.846
The environmental contribution of the product leads to improved recyclability within the production process.	0.822
Upgraded existing production equipment and processes	0.838
Increased investment in R&D of environmental protection technology	0.816
***Green service innovation* (Cronbach’s alpha = 0.947, *CR* = 0.947, AVE = 0.781)**	
The firm repackages existing products/services on the basis of its concern for the environment.	0.891
The firm frequently extends products/services on the basis of its concern for the environment	0.894
The firm creates and establishes new lines of products/services on the basis of its concern for the environment.	0.904
The firm offers new practices in new product/service development on the basis of its environmental concerns.	0.859
The firm proposes new practices in the promotion of new products/services related to environmental reputation.	0.869
***Supply chain risk* (Cronbach’s alpha = 0.957, *CR* = 0.957, AVE = 0.789)**	
Your supply chain is affected by external social risks.	0.801
Your supply chain is affected by risks related to your suppliers.	0.913
Your supply chain is affected by risks related to your customers.	0.924
Your supply chain is affected by external economic risks.	0.905
Your supply chain is affected by external environmental risks.	0.908
Your supply chain is affected by external political risks.	0.871
***Firm performance* (Cronbach’s alpha = 0.934, *CR* = 0.936, AVE = 0.745)**	
Profitability	0.769
Net profit margin	0.864
Profitability growth	0.907
Sales performance	0.855
Overall firm performance	0.912

*N = 337. AVE, average variance extracted; CR, composite reliability; FL, factor loading.*

*Model Summary: χ^2^(340) = 710.914, p < 0.001, CFI = 0.959, TLI = 0.954, IFI = 0.959, RMSEA = 0.057.*

**TABLE 2:** Descriptive statistics and correlations.

Variable	Mean	*SD*	1	2	3	4	5	6	7	8
1. Firm size	3.282	1.431	1.000							
2. Industry category	0.608	0.489	0.362**	1.000						
3. Ownership structure	0.620	0.486	−0.162**	–0.052	1.000					
4. Green product innovation	6.107	0.987	0.080	0.011	–0.024	***0.858***				
5. Green process innovation	6.239	0.950	0.099	0.033	–0.028	0.651**	***0.807***			
6. Green service innovation	6.217	1.028	0.181**	0.134*	–0.036	0.438**	0.486**	***0.884***		
7. Supply chain risk	2.011	1.137	–0.064	0.009	0.077	−0.615**	−0.392**	−0.386**	***0.888***	
8. Firm performance	5.993	1.122	0.141**	0.122*	–0.032	0.605**	0.612**	0.543**	−0.546**	***0.863***

N = 337. Figures in italicized bold denote the square root of the AVE of each study construct. *p < 0.05, **p < 0.01.

### Hypothesis Testing

Following the measure reliability and validity assessment, we empirically test the theoretical model and the hypotheses. Table 2 reports means, standard deviations, and correlations for each of the measures. Considering that no correlation is above the recommended level of 0.70 (Tabachnick and Fidell, 1996), multicollinearity is less likely to occur and threaten the interpretability of our results. Nonetheless, we checked for the potential presence of multicollinearity by examining the variance inflation factors (VIF) of each individual predictor in our regression model. The VIF values for all our individual predictors were all well below 10 (with the maximum being 4.02), suggesting that multicollinearity is less likely to be a problem in our analysis (Neter et al., 1996). To further mitigate multicollinearity concerns, we mean-centered all independent and moderating variables when running all interaction models (Aiken and West, 1991).

To test our hypotheses, we employed moderated hierarchical regression analysis. Table 3 presents the results of the moderated hierarchical regression analysis in which the changes in *R*-squared (Δ*R*^2^) at each step and standardized coefficients are reported. Model 1 included all control and independent variables. Models 2, 3, and 4 tested the interaction terms by introducing them individually. As shown in Model 1 of Table 3, the coefficients for the green product innovation (β = 0.159, *p* < 0.001), green process innovation (β = 0.302, *p* < 0.001), and green service innovation (β = 0.218, *p* < 0.001), were all positive and statistically significant. Therefore, Hypotheses 1, 2, and 3, which predicted a positive relationship between type of green innovation (i.e., green product innovation, green process innovation, and green service innovation) and firm performance, were all supported. Hypotheses 4a, 4b, and 4c proposed a negative moderating effect for supply chain risk on the relationship between type of green innovation and firm performance. The coefficient for the interaction term between green product innovation and supply chain risk in Model 2 (GTISCU) was negative and significant (β = −0.172, *p* < 0.001). It provides evidence that the relationship between green product innovation and firm performance is negatively moderated by supply chain risk, thereby supporting Hypothesis 4a. Similarly, as shown in Model 3 of Table 3, the results indicate the negative and also statistically significant interaction effect of green process innovation and supply chain risk (β = −0.130, *p* < 0.01). Accordingly, Hypothesis 4b was also supported. Conversely, the coefficient for the interaction term between green service innovation and firm performance (GEISCU) in Model 4 was negative but statistically insignificant (β = −0.014, n.s.). Therefore, Hypothesis 4c was not supported. In the following section, we discuss these results and their implications.

**TABLE 3:** Results of hierarchical regression analysis.

Variable	Model 1	Model 2	Model 3	Model 4
Firm size (annual sales)	0.018	0.028	0.028	0.018
Industry dummy	0.077	0.076	0.074	0.077
Ownership structure	0.014	0.027	0.027	0.014
Green product innovation (GTI)	0.159**	0.204***	0.152**	0.158**
Green process innovation (GSI)	0.302***	0.302***	0.337***	0.305***
Green service innovation (GEI)	0.218***	0.210***	0.228***	0.221***
Supply chain risk (SCR)	−0.247***	−0.288***	−0.259***	−0.248***
GTI SCR		−0.172***		
GSI SCR			−0.130**	
GEI SCR				–0.014
F statistics	56.447***	54.224***	52.300***	49.277***
*R* ^2^	0.546	0.569	0.561	0.546
Δ*R*^2^		0.024***	0.015**	0.000

N = 337. **p < 0.01, ***p < 0.001.

## Discussion and Conclusion

### Discussion and Implications for Theory and Practice

In this study, we theorized and empirically investigated the effect of various types of green innovation strategies on the firm performance of manufacturing firms in one of the world’s largest emerging economies (i.e., China). We have outlined the gains from green innovation and the context required for firms to leverage these gains. When environmental problems become serious enough to threaten human survival, the necessity and urgency of firms’ green innovation are further highlighted. Implementing green innovation has become a strategic choice for firms to grasp the development initiative in the post-pandemic era. This study is based on the circular economy in view of the international situation that multiple uncertainties of global supply chain risks have brought testing firms’ green innovation. Green innovation strategy is subdivided into green product innovation, green process innovation, and green service innovation. This study examines the different effects of these innovations on firm performance, thereby laying a theoretical foundation for firms to implement green innovation strategy in the post-pandemic era and help firms figure out how to implement green innovation to improve firm performance. At the same time, this study takes supply chain risk as a moderating variable and develops a comprehensive model to provide a useful reference for firms to avoid supply chain risk and accelerate green innovation strategy in the post-pandemic era. This study is expected to provide effective solutions for firms to implement green innovation strategy and improve firm performance.

By theorizing and offering empirical evidence of the importance of green innovation and supply chain risks, this study contributes to the literature in the following ways. First, green product innovation can significantly promote the performance of firms. Previous literature emphasized that green product innovation can help firms obtain market competitiveness (Chen et al., 2006). The current study extends this idea on the basis of the original literature. Given the positive impact of green product innovation on firm performance, firms should avoid using compounds that pollute the environment in product design. Designing environmentally friendly packaging for products is necessary.

Second, this study provides evidence that green process innovation has a positive effect on firm performance. Previous literature emphasized that green process innovation can solve the environmental problems in the process of production and consumption and improve the sustainability of production (Wang et al., 2021). This study further deepens and extends the previous research results. Given that green process innovation has a positive impact on firm performance, firms are suggested to avoid the use of harmful substances in the process of generating products; reduce the pollution of soil, water quality, air, and other environmental factors caused by the production of goods; adopt advanced environmental protection technology; and introduce pollution control equipment to make wastewater and waste materials recyclable to maximize energy efficiency. Through these green process innovation measures, firm performance will be largely improved.

Third, this study finds that green service innovation can significantly promote firm performance. This result is consistent with previous research results. In previous literature, green service innovation can make copying and gaining a competitive advantage difficult for competitors (Lin and Chen, 2017). It can also effectively help firms achieve sustainable development goals (Chen et al., 2015). The present study suggests that firms should pay attention to environmental issues. With the view to solving environmental problems, firms must redefine their existing products and services, provide environmental protection services for customers, and advocate the use of environmental protection sales methods and after-sales services, all of which will directly promote firm performance.

Fourth, for the moderating effect of supply chain risk, this study finds that supply chain risk plays a negative moderating role between green product innovation and firm performance. In the supply chain where firms generate products and deliver them to consumers, production networks such as raw material supply, parts manufacturing, labor supply, and logistics are closely linked. Mistakes in any link will bring challenges and risks to the main participants in the supply chain. In particular, implementing green product innovation by firms will inevitably require the production of products with environmentally friendly materials. Firms need to bear the risks brought by the replacement of raw materials (Cao and Zhang, 2011). According to the results of this research, when implementing the green innovation strategy, firms must pay close attention to the political, social, and environmental risk factors of each firm in the supply chain. They should also prepare response plans according to the changes in the situation, give full play to the positive role of green product innovation, and support the steady rise of firm performance.

Furthermore, this study finds that supply chain risk plays a negative moderating role between green process innovation and firm performance. For green process innovation, firms need to introduce environmental protection production machinery and equipment and replace suppliers. If the replaced machinery and equipment are from abroad, firms may need to bear transportation and political risks (Sheffi, 2001; Hendayani et al., 2021). In view of this possible scenario, firms should fully consider the cost burden triggered by replacing the machinery, equipment, and suppliers when carrying out green process innovation to avoid supply chain risk as much as possible. Finally, this study finds that supply chain risk does not play an obvious negative moderating role between green service innovation and firm performance. A plausible explanation for the insignificant moderating effect of supply chain risk on the relationship between green service innovation and firm performance is that green service innovation usually focuses on the service field which is performed by using environmentally friendly packing service and after-sales services. Such green service innovations are generally carried out by the firms themselves. In addition, other supply-chain related firms may be less likely to bring significant effects on the internal service innovation activities of such firms. In other words, the green service innovation of firms to improve performance will not be negatively affected by supply chain risk. Therefore, firms can prioritize the implementation of green service innovation according to their own situation. After the firm is strong enough to bear the risks brought by the supply chain, they can consider trying green product innovation and green process innovation, actively promote service measures related to environmental problems, and conquer consumers with green services.

### Limitations and Future Research Directions

Similar to all studies, this study is not without limitations. First, as our sample primarily comprised the small- and medium-sized firms in the Chinese economy, generating our findings to very large firms may be difficult. Thus, an important avenue for fruitful research is to incorporate large Chinese firms. Second, the sample of our study was limited to Chinese firms, and our focus on firms in China may raise some concerns on the generalizability of our findings. As competitive and institutional environments are heterogeneous and more importantly, cultures and policies may also vary significantly across emerging economies (Hoskisson et al., 2000), firms in these economies may not only have very different motivations and capability to pursue green innovation but also experience different degrees of supply chain risks. Therefore, future research is encouraged to replicate and extend our research focusing on China by employing comparative analysis and examining the role of green innovation and supply chain risks across emerging economies. Third, considering the complexity of the international situation and investigation, this study incorporated only a moderating variable of supply chain risk into the model. Future research can also examine the role of other internal organizational characteristics and external environmental variables in moderating or mediating the contribution of different types of green innovation to firm performance.

## Data Availability Statement

The raw data supporting the conclusions of this article will be made available by the authors, without undue reservation.

## Ethics Statement

Ethical review and approval was not required for the study on human participants in accordance with the local legislation and institutional requirements. Written informed consent from the patients/participants was not required to participate in this study in accordance with the national legislation and the institutional requirements.

## Author Contributions

Both authors listed have made a substantial, direct, and intellectual contribution to the work, and approved it for publication.

## Conflict of Interest

The authors declare that the research was conducted in the absence of any commercial or financial relationships that could be construed as a potential conflict of interest.

## Publisher’s Note

All claims expressed in this article are solely those of the authors and do not necessarily represent those of their affiliated organizations, or those of the publisher, the editors and the reviewers. Any product that may be evaluated in this article, or claim that may be made by its manufacturer, is not guaranteed or endorsed by the publisher.
